# Acquired and hereditary forms of recurrent angioedema: Update of treatment 

**DOI:** 10.5414/ALX1561E

**Published:** 2018-09-01

**Authors:** K. Bork

**Affiliations:** Universitäts-Hautklinik, Johannes-Gutenberg-Universität, Mainz

**Keywords:** angioedema, hereditary angioedema, C1 esterase inhibitor deficiency, bradykinin, C1-INH concentrate, bradykinin B2 receptor antagonist

## Abstract

The aim of treatment of hereditary angioedema (HAE) due to C1 esterase inhibitor deficiency (HAE-C1-INH) is either treating acute attacks or preventing attacks by using prophylactic treatment. For treating acute attacks, plasma-derived C1 inhibitor (C1-INH) concentrates, a bradykinin B2 receptor antagonist, and a recombinant human C1-INH are available in Europe. In the United States, a plasma-derived C1-INH concentrate, a bradykinin B2 receptor antagonist, and a plasma kallikrein inhibitor were approved for the treatment of acute attacks. Fresh frozen plasma is also available for treating acute attacks. Short-term prophylactic treatment focuses on C1-INH and attenuated androgens. Long-term prophylactic treatments include attenuated androgens such as danazol, stanozolol, and oxandrolone, antifibrinolytics, and a plasma-derived C1-INH concentrate. Plasma-derived C1-INH and a bradykinin B2 receptor antagonist are admitted for self-administration and home therapy. So the number of management options increased considerably within the last few years thus helping to diminish the burden of HAE.

**German version published in Allergologie, Vol. 36, No. 3/2013, pp. 108-119**

Angioedemas (former terms: angioneurotic edema, Quincke’s edema) are demarcated edemas that last for 1 – 7 days and recur at irregular intervals. Angioedemas manifest on the skin, more rarely on the tongue, glottis or larynx, gastrointestinal tract, and, very rarely, other soft tissue organs. The same clinical symptom “angioedema” belongs to various disease entities (Table 1). Most frequently, angioedemas are part of or equivalent to urticaria. Angioedemas are edemas of the subcutaneous tissue, and hives are edemas of the dermis. Thus, both have to be considered manifestations of a common underlying pathomechanism at different locations (“histamine-mediated angioedemas”). Recurrent angioedemas are an entirely different disease with regard to pathogenesis, clinical picture, and therapy. Probably, kinins, and among them mainly bradykinin, are involved in the development (“kinin-mediated angioedemas”). Some of these forms of angioedemas are based on a hereditary or acquired C1 inhibitor deficiency in the complement system. For other forms, pathogenesis has not been elucidated yet, but as antihistamines are not effective, they are counted among kinin-mediated angioedemas. These are angioedemas caused by ACE inhibitors, some idiopathic angioedemas, and hereditary angioedema with normal C1-INH (HAE type III). 

In Germany, thousands of patients suffer from a form of recurrent angioedema. There have even been some cases of death due to suffocation [22]. 

## Hereditary angioedema caused by C1 inhibitor deficiency 

### General overview

One of the most important forms of angioedema is the hereditary angioedema (HAE) caused by C1 esterase inhibitor (C1-INH) deficiency. Numerous cases of asphyxia have been described of this form of the disease, and the quality of life of many patients is impaired due to frequent edematous attacks. 

For this disease, almost all steps of pathogenesis from the underlying genetic defect to the clinical symptom “angioedema” could be elucidated. Apart from the traditional therapy options, there are new options that act at various steps of pathogenesis. 

The prevalence of HAE caused by C1-INH deficiency (HAE-C1-INH) is estimated to be around 1 : 50,000 [1]. In Germany, approximately 2,000 patients have been diagnosed. As far as we know, the disease is distributed equally between both genders, but the symptoms are usually more pronounced in women [19]. 

HAE-C1-INH is inherited in an autosomal dominant pattern. The gene encoding for the C1 esterase inhibitor (C1-INH) is located on the long arm of chromosome 11 in the sub-region q12-q13.1 and consists of 8 exons and 7 introns. New techniques have led to the detection of numerous mutations – more than 200 to date [40, 50]. Patients with type I HAE (85% of patients) possess one normally expressed C1-INH gene and one abnormal or deleted gene that is not expressed. Patients with type II HAE also possess one normal gene, the other gene is abnormal and expressed, which leads to the synthesis of a dysfunctional C1-INH. Type II HAE (15% of patients) develops due to point mutations in the C1-INH gene. New mutations are present in ~ 20% of patients. 

The C1-INH (Table 2) controls the spontaneous auto-activation of the first complement component (C1) as well as activated C1. A deficiency of C1-INH leads to the activation of the initial phase of the complement system and thus to a persisting reduction of the complement factor 4 (C4) in the plasma. Today, it is known that not the inhibitory effect of C1-INH on the complement system, but its inhibitory effect on the kinin-kallikrein system plays the most important role in the pathogenesis of HAE-C1-INH. The C1-INH is responsible for the inhibition of the major part of the plasma kallikrein and factor XIIa and thus is the most important regulator of kinin-kallikrein system activation. In acute HAE attacks, kallikrein is not sufficiently inhibited due to the C1-INH deficiency. This leads to the activation of the kinin-kallikrein system (contact system). At the end of the cascade, an increased amount of bradykinin is generated, which is the main mediator of increased vascular permeability and of edemas in HAE-C1-INH. 

Clinically, HAE-C1-INH is characterized by relapsing swelling of the skin (limbs, face, genitals), gastrointestinal episodes (painful abdominal cramps, possibly circulatory symptoms, vomiting, diarrhea), and edemas of the larynx and other organs [9, 19, 23]. Fatalities [9, 22] tend to affect patients with undiagnosed disease. 

### Patient treatment and management

HAE-C1-INH is a complex disease, which, if present in a more severe form, affects patients’ lives in various ways. Due to the nature of this disease, many patients need to take drugs for many years, decades, or even for their entire lives. Therapy aims at (a) avoidance of asphyxia and (b) alleviation of symptoms. As sudden (within hours) asphyxia can occur at any age (see above) and almost always starts without warning, the treatment of patients entails a high responsibility. It is essential to explicitly inform the patient about possible symptoms, particularly the initial symptoms of laryngeal edema (including globus sensation, difficulties swallowing, changes of the voice, onset of dyspnea). Furthermore, the patient should know what to do when these symptoms occur. Likewise, family members need to be informed about the disease and the measures they can take. As all this is important and time-consuming, it is recommendable to use the experience of an HAE treatment center. Ideally, HAE patients are managed by a nearby general practitioner or by their attending physician in cooperation with an HAE treatment center.[Table Table3]

### Treatment of acute edema attacks

**Indication**

Mild swelling of the hands and feet does not necessarily require treatment, for instance, when only the back of the hand is affected. Treatment is necessary if the swelling affects large areas or leads to functional impairments, e.g., swelling of an entire limb, or if the swelling in one region is usually followed by swelling in other body regions. Facial swelling in HAE should always be treated as it is frequently followed by laryngeal edema [19]. In mild cases of abdominal attacks, treatment with anticonvulsant suppositories (butylscopolammonium bromide-containing) may be sufficient. However, most abdominal attacks are so painful that treatment with C1-INH concentrate, icatibant, or recombinant C1-INH is necessary. Patients with hereditary angioedema in the head region and edema of the pharynx or larynx are emergency cases due to the risk of suffocation and should be immediately admitted to hospital. Treatment of laryngeal edema in HAE patients is based on how far the laryngeal edema has progressed. If life-threatening dyspnea is present, the patient should immediately be intubated, also using fiber-optic intubation, or – in cases of extreme emergency – cricothyrotomy or tracheotomy should be carried out. In any case, the drug therapy of choice is immediate treatment with a C1-INH preparation or icatibant. Corticosteroids, antihistamines, epinephrine, or epinephrine derivatives are not effective in cases of HAE caused by C1-INH deficiency! 

### Discontinuation of drugs that aggravate the disease

Estrogens (oral contraceptives, hormone replacement therapy) and ACE inhibitors can significantly increase the frequency and severity of HAE attacks. They should be discontinued and avoided in the future. 

**C1-INH concentrate **

*Efficacy*

Administration of C1-INH concentrate (i.e., of the protein the patients lack or which is not sufficiently functional) during an acute attack leads to the inhibition of the excessive kinin-kallikrein cascade (see above). 

Human C1-INH concentrate has proven highly effective in the treatment of acute attacks. In Germany, such a concentrate was first authorized in 1979 as “C1-Inaktivator Behringwerke”, then, in 1985, in a pasteurized, virus-inactive form as “C1-Inaktivator Behring”, in 2001 as “Berinert P” (CSL Behring GmbH, Marburg, Germany), and since 2011 it has also been available as (pasteurized, nanofiltered) “Berinert” for intravenous injection. Thus, the medical community in Germany has more than 30 years of experience in treating acute HAE attacks with this concentrate [5]. In a series of non-placebo-controlled trials, its effectiveness in laryngeal edemas [8, 17], attacks of abdominal pain [18], and skin swellings [24] in HAE caused by C1-INH deficiency was demonstrated. 

In 1996, a randomized, double-blind, placebo-controlled trial was published in which the efficacy of a vapor-heated C1-INH concentrate manufactured by Baxter/Immuno was evaluated [55]. The time to first improvement was 55 minutes in 22 patients with acute HAE attacks in all localizations, compared to 563 minutes in patients treated with placebo. Despite proven efficacy, the C1-INH concentrate was taken off the market by the manufacturer in 2003. During the course of marketing authorization of Berinert in the USA, a randomized, double-blind, placebo-controlled trial was carried out from 2005 to 2007. It included 125 patients with type I or type II HAE (I.M.P.A.C.T1), and a statistically significant superiority of Berinert (20 U per kg body weight (BW)) as compared to placebo was shown [32]. In 2008, this study led to the marketing authorization of the treatment of acute attacks using weight-adjusted doses in many European countries (children and adults receive the same dosage). At this dosage, symptom relief was achieved after 30 minutes, independently of the severity of the edema in the face or abdominal region. The rebound phenomenon, i.e., the recurrence of swelling after injection, has not been observed in C1-INH concentrate therapy. 

In June 2011, the C1-INH concentrate Cinryze (ViroPharma Inc., USA) was also authorized in Europe for the treatment of acute attacks. 

*Dosage*

In the above-mentioned double-blind study [32], at a dosage of 20 U per kg BW, Berinert was significantly more effective in treating acute attacks in the face and abdominal region than placebo. Treatment with 10 U per kg BW was more effective than placebo, but the difference did not reach statistical significance. This result does not correspond with other published data that were derived from thousands of patients treated with C1-INH concentrate, including those participating in a randomized double-blind trial in the USA in 1996 [55]. According to those studies, a dosage of 500 U (corresponding to 7.1 mg/kg BW in a patient weighing 70 kg) was highly effective in the vast majority of patients. For treatment of some attacks and patients, 1,000 U (14.2 mg/kg BW) were necessary, but almost never more. In an observational study in 61 patients, 500 U Berinert P were necessary in 468 attacks, 1,000 U were necessary in only 9 attacks (2%) [34]. In other studies, 500 U were sufficient to treat 68.6% of 4,834 abdominal attacks in 75 patients [18], 81% of 2,104 cases of skin swelling in 47 patients [24], and 24.9% of 193 laryngeal edemas in 18 patients [8]. 

It is unclear why the I.M.P.A.C.T.1 results are inconsistent with all other data. One reason might be the strong placebo effect in HAE attacks that were not treated with Berinert; this was also observed in other studies. Patients with abdominal attacks were hospitalized and the study environment did not correspond to “daily life”. In addition, the study was partially conducted in the USA, where there was no previous experience with therapy of acute attacks (except therapy with fresh frozen plasma). The results of double-blind, placebo-controlled, randomized trials have the highest level of evidence. In Australia, Canada, USA, and many European countries, the results of the randomized double-blind trial published in 2009 led to the marketing authorization of the 20 U/kg BW, i.e., 1,400 U (almost 3 ampoules of Berinert in a patient weighing 70 kg) dosage for the treatment of acute attacks. 

Due to their potentially life-threatening nature, laryngeal edemas should always be treated with a Berinert dose of 20 U/kg BW. In cases of non-life-threatening swelling, i.e., skin swelling or abdominal attacks, which account for ~ 99% of acute attacks, it is justifiable to adjust the dosage to the patient’s needs and choose 500 U or, if necessary, 1,000 U per attack. 

For Cinryze, the manufacturer recommends a dose of 1,000 U for the treatment of an acute attack in adolescents and adults.

*Safety*

Data on the safety of one of the available preparations (Berinert) are based on more than 35 years of experience. The safety profile has proven extremely positive. Adverse events are very rare (in < 1/1,000 applications) and include allergic anaphylactic reactions and/or rise of body temperature. There have been some reports of unexpected, slow increases of attack frequency and other signs of intensified disease activity after long-term and frequent use of C1-INH concentrate [15, 16]. Cases of virus transmission were not observed. 

As for all patients receiving plasma derivatives, vaccination against hepatitis A and B is recommended, particularly when C1-INH concentrate is administered frequently and regularly. 

*Time of injection*

Treatment should take place as soon as possible during an attack [18]. In a study examining more than 2,700 abdominal attacks, it was shown that early injection was more effective than later injection and that lower doses of the drug were needed [18]. First improvement is usually experienced within 30 – 60 minutes after IV injection. 

*Self-administration, self-treatment at home*

After training on IV injection techniques, some patients inject Berinert themselves, or injections are applied by their family members (self-treatment at home) [44, 46, 54]. Berinert was authorized for self-administration by the Paul-Ehrlich-Institut in August 2011. In June 2011, the C1-INH concentrate Cinryze was also authorized in Europe for self-administration. However, even if patients treat themselves or are treated by their family members at home, the attending physician still bears the responsibility. 

**Icatibant**

Icatibant is a bradykinin-B2-receptor antagonist with structure similar to bradykinin. By binding of bradykinin to the receptor, an acute HAE attack due to C1-INH deficiency can be treated [7]. In several (controlled [12] and uncontrolled [28, 47]) studies, subcutaneously injectable icatibant (dose 30 mg) has been proven safe and effective in acute attacks of HAE-C1-INH. A first subjective symptom improvement was achieved after an average of 48 minutes, the first clinically relevant improvement was observed after a median of 2 hours. Adverse events included reactions at the injection site, like redness, wheals, or pain (in > 1/10 applications), and some other non-severe adverse events, like nausea, abdominal pain, or congested nose (in < 1/10 and > 1/100 applications). According to the FAST-1 and FAST-2 studies, the swelling recurred 6 or more hours after injection (rebound effect) in ~ 10% of treated attacks, which made a second or third injection with icatibant necessary. For the treatment of HAE attacks in adults, icatibant has been available in the European Union since July 2008, and in the USA since 2011. 

**Conestat alpha**

*Efficacy*

Conestat alpha is a recombinant human (rh) C1 inhibitor (Ruconest/Rhucin, Pharming Group NV, The Netherlands) developed for the treatment of HAE attacks. The rhC1-INH is produced in the mammary glands of transgenic rabbits by recombinant DNA technology. Clinical studies have demonstrated the high effectiveness of rhC1-INH in the treatment of acute HAE attacks [58]. Differences in the post-translational glycosylation result in a significantly reduced half-life (~ 2 hours) compared to C1-INH in human plasma. In October 2010, Ruconest was authorized in Europe for the treatment of HAE attacks in adults. 

*Dosage*

Ruconest is injected intravenously. According to the manufacturer, the dosage for adults with up to 84 kg BW is 50 U/kg BW, adults with a higher BW receive 4,200 Units (2 ampoules). 

*Safety*

The most frequent adverse event of Ruconest is headache. One healthy control subject with an undisclosed rabbit allergy developed an anaphylactic reaction after the administration of rhC1-INH. Therefore, the European Medicines Agency decided that Ruconest is contraindicated in patients with known or suspected rabbit allergy or with positive IgE against rabbit allergens due to the risk of allergic reactions. The IgE antibody test should be repeated after 10 treatments or at least once a year. 

**Fresh frozen plasma**

Because it contains C1-INH, fresh frozen plasma (FFP) is effective in the treatment of acute HAE attacks caused by C1-INH deficiency. No controlled studies are available, only several case observations. However, FFP not only contains coagulation factors, but also proteins of the kinin-kallikrein system, which could result in an increased bradykinin production. This could lead to an aggravation of the acute attack in some patients [51]. FFP is not virus-inactivated, however, virus-inactivated preparations are also available. Due to these and further disadvantages, the use of FFP is not recommended for HAE treatment in Germany, where C1-INH concentrates, icatibant, and recombinant C1-INH are available. 

### Drugs for long-term therapy for prevention of edema attacks

The treatment of acute attacks should be preferred over long-term prevention [29]. Long-term prevention should be considered if the patient suffers from frequent attacks, e.g., if despite the best possible treatment of acute attacks, > 12 severe attacks per year or > 24 days with HAE symptoms occur. 

**Attenuated androgens**

Androgen derivatives, in particular, danazol, stanozolol, and oxandrolone, have successfully been used for long-term prevention. The indication for long-term prevention using drugs has to consider the risks of androgens and the individual situation of each patient. Androgens are highly effective. In a double-blind, randomized cross-over study investigating danazol (600 mg daily) vs. placebo, danazol significantly reduced the number of attacks (2.2% vs. 93.6%) [39]. However, such a high danazol dose is no longer recommended. In a study published in 2008, 46% of patients were completely symptom-free under danazol or had 1 or less attack per year [11]. The average yearly frequency of attacks was 33.3 before and 5.4 during danazol treatment. The attacks were markedly milder under danazol treatment than before or after the therapy. Not all patients respond to treatment with androgens. In some patients, the treatment can become less effective after several years [37]. 

Nevertheless, attenuated androgens can cause adverse events that should not be underestimated; benefit and risk should be weighed carefully. Adverse events include, among many others, weight increase (~ 40% of treated patients), menstruation disturbances (~ 30% of treated women), and virilization in female patients as well as hepatotoxicity, depression, and arterial hypertension when used over longer periods of time [11, 30, 60]. Liver cell adenomas have been observed [20, 21] as well as liver cell carcinomas in 2 patients. Regular controls of the liver function and ultrasound imaging of the liver are necessary as are controls regarding the other possible adverse events [11]. Adverse events are dose-dependent [11]. 

The androgens indicated above are not authorized for use in HAE in Germany and have to be obtained in other countries. Physicians who carry out androgen treatment in HAE patients are responsible for closely monitoring the patient with regard to adverse events. 

Possible indications for attenuated androgens are particularly frequent attacks (more than 1 – 2 per month) or multiple laryngeal edemas. 

For these reasons, and because androgens have been used with the wrong indications and in too high doses, with the corresponding consequences, it is recommended to start this kind of therapy in an HAE treatment center. 

The dosage of danazol is 200 mg or less per day. The dose should be adjusted individually for each patient so that the lowest possible dose leading to symptom suppression is reached. 

This kind of treatment should not be carried out in children, pregnant or lactating women, or in patients with prostate carcinoma. 

**Tranexamic acid**

Two antifibrinolytic agents have been proven effective in HAE: epsilon aminocaproic acid (EACA) and tranexamic acid. A double-blind placebo-controlled cross-over study using 16 g EACA daily vs. placebo showed a significant efficacy of EACA in 4 patients [35]. In a further placebo-controlled cross-over study using tranexamic acid, a significant HAE improvement through tranexamic acid was shown in most patients [53]. Since 1972, tranexamic acid has been used for long-term treatment of HAE [4]. It is tolerated better than EACA. The efficacy of tranexamic acid in adults is usually markedly lower than that of attenuated androgens, but due to its milder adverse events, it is frequently used in children with HAE. As tranexamic acid is an antifibrinolytic agent, thromboembolic events can occur. Patients with thrombophilia should not be treated with tranexamic acid. Furthermore, color vision can be disturbed so that the fundus of the eye should be controlled regularly. 

*Dosage*

Start of therapy with 20 – 50 mg/kg BW to a maximum of 3 g in adults. The daily dose should be down-titrated to the lowest effective dose. 

*Contraindications*

Pregnancy, renal disease, acute thrombosis, or thromboembolic events. Strongly limited use when family history of thrombophilia or active thromboembolic events is present. 

**C1-INH concentrate**

C1-INH concentrate can be used for long-term prevention. The first HAE patient received this treatment in 1989 – with considerable success [25]. Further studies have shown good results with regard to efficacy and tolerability [14, 45, 55]. The weekly dose is 2-times 500 U C1-INH concentrate or more. In a double-blind, placebo-controlled cross-over study (22 patients in two 12-week periods), the nanofiltered C1-INH concentrate Cinryze (ViroPharma Inc., USA), in a dose of 2-times 1,000 U/week, reduced the number of HAE attacks from 12.7 to 6.3 [59]. Cinryze was authorized for long-term prophylaxis in the USA in October 2008 and in Europe in June 2011. 

In some of the patients who receive frequent injections of C1-INH concentrate, also under long-term prevention treatment, the disease activity increases: attacks occur more frequently, more C1-INH concentrate is needed, and/or fast-developing multi-site attacks occur [16]. 

## Hereditary angioedema with normal C1-INH 

Type III HAE is another familial form of angioedemas. The patients (almost exclusively women) have normal plasma C1-INH [10, 13]. Estrogens, i.e., oral contraceptives, pregnancy, and hormonal replacement therapy, often play an important role as triggering and aggravating factors. In some patients, mutations in the factor XII gene have been demonstrated [26, 33]. Due to the rareness of the disease, therapeutic experience with type III HAE is scarce. In acute attacks, C1-INH concentrate and icatibant have proven effective. For long-term prevention, progesterone, tranexamic acid, and danazol have successfully been used in individual cases [6]. 

## Angioedemas caused by acquired C1-INH deficiency 

In some patients with angioedema, C1-INH deficiency is caused by an increased C1-INH catabolism. As a result, C1q is usually decreased. The symptoms correspond to HAE-C1-INH [57]. In many of these patients, underlying B-cell dysfunctions are present, e.g., monoclonal gammopathy of unknown significance or malignant lymphomas, and quite frequently, these are detected in the course of diagnostic work-up for angioedema. In some of these patients, inhibiting autoantibodies against C1-INH can be detected [2]. This disease is about 10-times more rare than HAE-C1-INH. For therapy, C1-INH concentrate and icatibant have been used successfully [31]. 

## Angioedemas caused by ACE inhibitors 

ACE inhibitors lead to recurrent angioedemas in approximately 0.1 – 2.2% of users; frequently, the face or the tongue are affected [27, 48, 52]. 

The prevalence is even higher in patients of African descent. Because of the extremely high consumption of ACE inhibitors, angioedemas due to ACE inhibitors have become frequent in Germany. Numerous study results suggest that bradykinin is responsible for the development of angioedemas caused by ACE inhibitors. The exact pathomechanism is still unknown. Clinical symptoms include facial swelling, tongue swelling, and, more rarely, painful abdominal attacks. Upper airway obstructions are relatively frequent, and even some deaths due to suffocation have been reported. The period of time between start of medication and onset of the first angioedema can vary from months and several years so that the association between angioedema and ACE inhibitors is sometimes only recognized quite late. 

In cases of progressed laryngeal edema or tongue swelling, emergency measures to keep the upper airways open can become necessary. It is essential to discontinue the ACE inhibitor in order to not endanger the patient. Treatment with corticosteroids or antihistamines is frequently not effective. Efficacy of C1-INH concentrate [49] and icatibant [3] has been reported in single cases. 

## Recurrent angioedemas in chronic urticaria 

More than 50% of patients with chronic recurrent urticaria (CRU) report the occasional or frequent occurrence of angioedemas [36]. Thus, angioedemas are an optional symptom of this disease. Due to the high prevalence of CRU (approximately 1 – 5% of the general population is affected), urticaria-associated angioedemas are particularly frequent [38, 42]. Angioedemas in CRU usually respond well to corticosteroids and antihistamines. 

## Recurrent idiopathic angioedemas 

These are angioedemas that cannot be classified among any of the other forms; thus, diagnosis is made by exclusion. This means that these patients do not have a family history of angioedema, no C1-INH deficiency is present, and the angioedema cannot be traced back to drugs or other triggering factors. Knowledge of pathogenesis and therapy of this form of angioedema is still scarce [36, 56]. In some of these patients, antihistamines and corticosteroids are effective. Other patients do not respond to this kind of therapy. 

## Angioedemas in allergic or pseudo-allergic reactions 

Angioedemas, mostly in the form of facial swelling, also occur as a symptom of acute allergic or pseudo-allergic reactions [41, 56]. In these cases, they are frequently, but not always, associated with urticaria or, more rarely, with the symptoms of an anaphylactic shock. Mostly, this is a unique event, which only recurs at re-exposure. Triggering factors are mainly food, drugs, and insect stings. It is also well known that aspirin can lead to angioedemas. Corticosteroids and antihistamines are usually effective in this form of angioedema. 

## Course of action in cases of angioedema 

1. If an angioedema patient presents in the interval between two events, the time until the next event should be used for diagnostic work-up so that the nature of the disease can be classified. This helps to choose an adequate therapy when the next angioedema attack occurs. Incorrect therapy could lead to life-threatening situations. 

2. If a patient presents with acute angioedema, it has to be assessed as to how threatening the symptoms are. Laryngeal edemas and tongue edemas are potentially life threatening. Acute life-threatening events with high-grade dyspnea require emergency measures to keep the airways open (intubation; if necessary, percutaneous puncture of the trachea or cricothyroid ligament; in emergency, coniotomy or tracheotomy). 

Swelling of the face or lips alone is not threatening, but it should be taken into account that facial swelling can be followed by laryngeal edema. Swelling of the limbs is not life threatening either, nor are painful abdominal attacks. 

The drug therapy varies according to the form of angioedema. Therefore, it should be assessed as to whether a certain diagnosis has already been made. Patients with HAE-C1-INH usually have an emergency health card containing information on their therapy. It is essential to take into account all these pieces of information. The author has heard of numerous cases of suffocation or “near suffocations”, particularly in patients with HAE-C1-INH, which could be traced back to health personnel ignoring information on diagnosis and therapy provided by the patient. It is important to note that cortisone and antihistamines are clearly ineffective in some types of angioedemas (HAE-C1-INH, HAE with normal C1-INH, angioedemas caused by acquired C1-INH deficiency, angioedemas caused by ACE inhibitors) and only questionably effective in others. If treatment is ineffective, valuable time can be lost that would be necessary to take emergency measures. 

If no information on patient history is available, a treatment attempt using high-dose corticosteroids and antihistamines is justified, but the patient has to be closely followed. After the acute symptoms have resolved, a complete diagnostic work-up should be carried out (cf. point 1). 

The German Society for Angioedema Research (GSAR; founded in 1996) dedicates itself to the clinical issues and research of angioedemas (www.angioedema.de). For patients with hereditary angioedema, a patient organization (www.schwellungen.de) exists. 

**Table 1.** Forms of angioedema with and without C1 inhibitor deficiency.

Hereditary angioedema caused by C1 inhibitor deficiency
Type I (reduced activity and concentration of C1 inhibitor in the plasma)
Type II (reduced activity with normal or increased concentration of C1 inhibitor in the plasma)
Hereditary angioedema with normal C1 inhibitor
Hereditary angioedema caused by factor XII gene mutations
Hereditary angioedema with unknown genetic background
Angioedemas caused by acquired C1 inhibitor deficiency
Angioedema caused by ACE inhibitor or other drugs
Recurrent angioedema in chronic urticarial
Recurrent idiopathic angioedema
Angioedemas in allergic or pseudo-allergic reactions

**Table 2.** C1 esterase inhibitor (C1-INH).

**Characteristics**
Glycoprotein
Single-chain
478 amino acids
Molecular weight: 105 kDa

**Site of formation**
Mainly generated in hepatocytes, to a small extent also in monocytes, macrophages, skin fibroblasts, and endothelial cells of the umbilical cord
Belongs to the SERPIN family of serin protease inhibitors

**Functions**
Main inhibitor of various proteases of the complement system (C1r, C1s, mannan-binding, lectin-associated serin protease (MASP)
Main inhibitor of kallikrein and factor XIIa, the proteases of the kinin-kallikrein system
Minor inhibitor of plasmin, a protease of the fibrinolytic system, and of factor Xia, a protease of the coagulation system

**Table 3. Table3:** Efficacy of various drugs in recurrent angioedema.

Drug	Histamine (mast cell)-mediated angioedema	Kinin-mediated angioedema
Antihistamines	+	0
Corticosteroids	+	0
C1-INH concentrate	0	+
Icatibant	0	+
Recombinant C1-INH	0	+
Danazol	0	+
Tranexamic acid	(+)	+
